# Neurocognitive impairment and its associated factors among patients with diabetes mellitus who have follow up at referral hospital in Northwest, Ethiopia

**DOI:** 10.3389/fendo.2024.1459585

**Published:** 2024-11-26

**Authors:** Arefaynie Simachew, Asmamaw Getnet, Fentahun Minwuyelet, Haymanot Zeleke Mitiku, Worku Misganaw Kebede, Fassikaw Kebede Bizuneh, Bekele Getenet Tiruneh, Dejen Tsegaye

**Affiliations:** ^1^ Debre Markos Comprehensive Specialized Hospital, Debre Markos, Amhara, Ethiopia; ^2^ Department of Nursing, College of Health and Medicine, Debre Markos University, Debre Marqos, Ethiopia; ^3^ Department of Public Health, College of Health and Medicine, Debre Markos University, Debre Markos, Ethiopia; ^4^ Department of Critical Care and Emergency Medicine, College of Health and Medicine, Debre Markos University, Debre Markos, Ethiopia

**Keywords:** prevalence, cognitive impairment, diabetes mellitus, Ethiopia, Northwest

## Abstract

**Background:**

Neurocognitive impairment is a condition that makes it difficult for a person to make decisions that affect memory, learning new things, concentration on daily activities, and can range from mild to severe forms. It is a major health problem, less known and less addressed complication of diabetes mellitus.

**Objectives:**

The aim of the study is to assess prevalence of neurocognitive impairment and associated factors among diabetic mellitus patients.

**Methods:**

We conducted an institutional-based cross-sectional study involving 512 diabetic patients under follow-up at XXX Specialized Hospital from March 1 to April 30, 2023. Data on cognition, behavior, and depression were collected using standardized tools, including the Mini Mental State Examination for cognition, the CAGE assessment tool for alcohol-related behavior, and the PHQ-9 for depression. These assessments were conducted through face-to-face interviews and chart reviews. A diagnosis of diabetes is confirmed in adults when fasting blood glucose levels exceed 126 mg/dl in three consecutive follow-up measurements. Data entry was performed using Epidata version 4.6, and analysis was conducted using SPSS version 26. Variables with a P-value < 0.25 in bivariate analysis were included in multivariable logistic regression. Statistical significance was set at P ≤ 0.05 with a 95% CI. Results were presented using tables, graphs, and descriptive text.

**Results:**

The prevalence of neurocognitive impairment among diabetic patients at XXX Comprehensive Specialized Hospital was 28.3% [95% CI: 24.57–32.39]. Factors associated with this impairment included being female (AOR=2.29 [95% CI: 1.43-3.67]), rural residence (AOR=3.16 [95% CI: 2.01-4.95]), comorbidity (AOR=3.30 [95% CI: 2.08-5.23]), diabetes duration of 6-10 years (AOR=1.72 [95% CI: 1.01-2.94]), diabetes duration >10 years, and blood sugar level >126 mg/dl (AOR=2.25 [95% CI: 1.42-3.57]). Patients are encouraged to adhere to proper medication regimens to effectively control their blood glucose levels. This study found a high prevalence of neurocognitive impairment (NCI) among diabetic patients, affecting about one-fourth based on MMSE scores. Key risk factors identified include female gender, rural residence, comorbidities, longer duration of diabetes, and elevated blood glucose levels.

## Background

Neurocognitive impairment (NCI) is a condition that hinders a person’s ability to make decisions related to memory, learning, concentration, and daily activities, and can range from mild to severe forms (1). Diabetes mellitus (DM), a chronic disease characterized by elevated blood sugar levels, affects various body systems, including the brain, potentially leading to neurocognitive impairment (2). The exact cause of cognitive impairment due to diabetes remains unknown but is likely multifactorial (3). Poorly controlled blood glucose levels may predispose individuals with diabetes to cognitive impairment (4, 5).

Potential mechanisms by which diabetes affects brain health include dysfunction of the inflammatory system, oxidative stress, and endothelial dysfunction. These factors contribute to insulin resistance and elevated blood glucose levels (6). Diabetic patients show a significantly higher incidence of reduced cerebral blood flow, cerebral atrophy, white matter disease, and cerebral microvascular disease compared to age-matched non-diabetic patients (7).

NCI can be categorized into subtypes based on severity: Delirium, Mild Cognitive Impairment, and Major Cognitive Impairment (8). Mild Cognitive Impairment represents the early stage of memory loss or other cognitive abilities, where individuals can still independently perform most daily activities. Major Neurocognitive Impairment involves multiple cognitive deficits severe enough to impair daily functioning, indicating a decline from a previous level of functioning (9, 10).

According to WHO estimates, the number of people with NCI is projected to increase from 7.8 million in 2013 to over 27 million by 2050 (11). Longitudinal studies indicate that the rate of cognitive decline in patients with DM is up to twice as fast as that associated with normal aging, and DM patients have an increased risk of NCI (12). A recent systematic review and meta-analysis found that the prevalence of NCI among DM patients could be as high as 45% (13). It is estimated that individuals with diabetes mellitus are 1.5 times more likely to experience NCIand dementia compared to healthy individuals (14).

NCI associated with DM is a major obstacle to effective treatment and complicating the long-term management of diabetic patients (15). This impairment also creates significant stress and challenges for caregivers, including physicians, case workers, nurses, and other providers (16).

NCI is also a major health issue in DM patients that affects a person’s ability to be independent and is an important determinant of quality of life (17, 18). The combined cost of healthcare and lost income due to diabetes-related NCI has already reached $81 billion annually and is projected to rise to $2 trillion by 2030 (9).

Cognitive decline occurs more frequently in patients with Type 2 diabetes mellitus, particularly in those with atrial fibrillation, compared to individuals without DM-2 and arterial fibrillation. The findings indicate that advanced age and dyslipidemia are significant risk factors for cognitive decline in patients with DM-2 (19).

Ethiopia is actively working to develop policies and strategies aimed at addressing mental health gaps, including neurocognitive impairment (NCI) associated with diabetes. Understanding cognitive function in diabetes is essential, as cognitive dysfunction can influence patient behaviors and clinical presentations, ultimately affecting self-care and diabetes management strategies. However, significant knowledge gaps persist regarding NCI in patients with diabetes mellitus, particularly concerning factors such as diabetes type, age, sex, medication side effects, duration of illness (1 to 5 years, 6 to 10 years, and more than 10 years) (20), residency, education level, body mass index [>25 Kg/m2 (21)] and glycemic control [≥126 mg/dl (21)].

Types of diabetes, including Type 1 and Type 2, have distinct pathophysiological mechanisms and long-term complications that can differentially affect cognitive function (22). Age is also a known risk factor for cognitive decline; older adults with diabetes may experience accelerated cognitive impairment due to age-related neurodegenerative processes compounded by metabolic dysregulation (23). Furthermore, sex differences can influence cognitive function and the prevalence of diabetes-related complications, making it essential to consider sex as a variable, as hormonal variations may affect brain health (24).

Additionally, medications used to manage diabetes can have side effects that impact cognitive performance, highlighting the importance of understanding these effects in assessing overall cognitive health (25). The length of morbidity plays a role as well; longer exposure to hyperglycemia and other metabolic disturbances may lead to more significant neurocognitive impairment (26). Residency, including urban versus rural living conditions, can affect access to healthcare, education, and lifestyle choices, all of which may influence cognitive health (27).

Education level is another factor; higher education levels are generally associated with better cognitive reserve, which may protect against cognitive decline, thereby assessing the potential impact of socioeconomic factors on cognitive performance (28). Finally, glycemic control is critical, as poor control is linked to cognitive impairment, with chronic hyperglycemia potentially leading to vascular damage and neuroinflammation, adversely affecting cognitive functions (29).

The absence of research on neurocognitive impairment (NCI) among patients with diabetes mellitus in this study area highlights a significant knowledge gap. Therefore, this study aims to address these gaps by assessing the prevalence of NCI and identifying its associated factors. Conducted as an institution-based cross-sectional study at a referral hospital in northwest Ethiopia, this research will provide valuable insights into the burden of NCI in diabetic patients within this specific population.

## Literature review

### Prevalence of neurocognitive impairment among diabetes mellitus patients

Various studies worldwide have explored the prevalence of neurocognitive impairment among diabetic patients, revealing that this condition is widespread. A global systematic review indicated a prevalence of cognitive impairment of 21.2% (30). In Europe and Asia, another systematic review found a pooled prevalence of mild cognitive impairment among diabetic patients of 45%, with rates of 82.3% in Europe and 98% in Asia (31).

Similarly, a cross-sectional study in India found a neurocognitive impairment prevalence of 33.73% (32), while another prospective study reported a rate of 24% (33). In Malaysia, the prevalence was 46.9% (34), and in Chile, it was 17.3% (35). A study in Saudi Arabia revealed an overall prevalence of 80.3%, with 33.8% classified as severe (36). In Egypt, the prevalence among diabetic patients was 34% (37), and in Nigeria, it was 40% (38). A study in Kenya reported a prevalence of 32% (1). In Ethiopia, various studies have examined neurocognitive impairment among diabetic patients, including a recent cross-sectional study in Bahir Dar city referral hospitals, which found a prevalence of 27.6% (20).

### Socio demographic factors related with neurocognitive impairment

Studies in Mexico, Nigeria, and India identified older age as a risk factor for neurocognitive impairment among diabetic patients (2, 38, 39). Additionally, research in Nigeria, India, and Chile found that being female was associated with increased risk (32, 35, 38). In Cameroon, Brazil, and India, low education levels were linked to neurocognitive impairment (2, 15, 40). A study in China highlighted that having diabetes for 6-10 years or more than 10 years was a significant risk factor (41). In India, low socioeconomic status was also associated with neurocognitive impairment (2). Furthermore, studies in Jimma and Bahir Dar identified farming as a significant risk factor, while research in Bahir Dar referral hospitals noted that rural residency was linked to increased risk of neurocognitive impairment (20, 42).

### Clinical related factors

A population-based prospective study in the USA found that poor glycemic control was linked to neurocognitive impairment (NCI) in diabetic patients (43). In the UK, hypoglycemia was identified as a higher risk factor for developing NCI (44). Studies in India, China, and Jimma demonstrated a significant association between hyperglycemia and NCI (32, 42, 45). A cross-sectional study in China showed that comorbidities were significantly associated with NCI among diabetic patients, a finding supported by a retrospective study in Brazil (40, 46). In both China and Jimma, the type of treatment modality was also identified as a significant risk factor (47). Additionally, cross-sectional studies in Saudi Arabia and China found that depression was significantly associated with NCI (48, 49). A study in Egypt indicated that a duration of diabetes exceeding 10 years was significantly linked to cognitive impairment (37).

### Behavioral related factors

A cross-sectional study involving 516 diabetic individuals in India found a strong association between cognitive impairment and smoking (P < 0.001) and alcohol consumption (P < 0.001) (32). A 2020 study in China revealed that smoking habits doubled the incidence of cognitive impairment compared to non-smokers (41). Similarly, research conducted in Saudi Arabia indicated that smokers were significantly associated with neurocognitive impairment (48).

## Methods

### Study setting and time

The study was conducted from March 1 to April 30, 2023, at XXX Specialized Hospital in Ethiopia. Located in Debre Markos town, it is situated 299 km northwest of Addis Ababa and 263 km from Bahir Dar. The hospital serves as a specialized facility for West East Gojjam, West Gojjam, and Awi Zone within the region, as well as other areas such as Oromia and Benishangul-Gumuz. It provides both inpatient and outpatient services and serves a population catchment area of 5,000,000. The hospital employs a total of 307 nurses, 4 internists, and 33 general practitioners. The outpatient department alone manages approximately 1,100 diabetes mellitus patients every two months (in the follow up clinic), making it a primary center for diabetes care in the region.

### Study design

Institution based cross-sectional study design was employed.

### Source population

The source population for this study were diabetes mellitus patients who were on chronic follow-up at XXX Specialize Hospital.

### Study population

The study population consisted of patients diagnosed with DM who were receiving follow-up care at XXX Specialized Hospital and were available during the data collection period. Patients were selected using a systematic random sampling technique.

### Inclusion criteria

All adult diabetes mellitus (DM) patients aged 18 and above, who attended DMCSH (Debre Markos Comprehensive Specialized Hospital) and had at least one follow-up visit during the study period, were included in the study.

### Exclusion criteria

Patients who were critically ill, already diagnosed with dementia, or had missing important variables such as blood glucose levels and blood pressure in their charts during the study period were excluded from the study.

### Sample size determination

Sample size was determined by using single population proportion formula considering the following assumptions: 95% confidence interval with 4% margin (0.04) of error and proportion of NCI among diabetic patients. Prevalence of NCI was 27.6% with 95% CI which was conducted in XXX referral hospitals (20).


$$\text{n}=\frac{{\text{Z}}^{2}.\text{P}(1-\text{P})}{{\text{d}}^{2}}$$

n= (1.96)2(0.276) (0.724)/(0.04)2 = 479 Where, n = sample size Z= confidence level (1.96) p= estimated prevalence (0.276) d= Margin of error to be tolerated (0.04). By adding 10% non-response rate, the final sample size was found to be 528.

### Dependent variable

Neurocognitive impairment was the dependent variable.

### Independent variables

Independent variables were Sociodemographic variables: age, sex, marital status educational level, occupation, and place of residence, Clinical related variables: Type of DM, Blood glucose level, types of treatment for diabetes, Body mass index, duration with diabetes and presence of comorbidities and Behavioral related variables: Chewing Chat, smoking, alcohol consumption.

### Operational definitions

#### Neurocognitive impairment

is the health disturbance in which the person’s ability of thinking, remembering, coping, judgmental ability, and orientation is become decline (20). A MMSE (Mini Mental State Examination) score of ≥ 25 point (out of 30) was considered as effectively normal (intact) cognition and ≤ 24 points was considered as NCI (20, 38).

#### Chat abuse/dependence

if an individual score of 2 or more points by CAGE (Cut down Annoyed Guilty Eye opener) assessment indicates likelihood of chat abuse, i.e., individual has chat use disorder (50).

#### Tobacco abuse/dependence

if an individual score of 2 or more points by CAGE assessment indicates likelihood of tobacco abuse, i.e., tobacco use disorder (51).

#### Alcohol abuse/dependence

if an individual score of 2 or more points by CAGE assessment indicates likelihood of alcohol abuse (52)

#### Depression

is a mood disorder that causes a persistent feeling of sadness and loss of interest. Total score of Depression scale is 27 points, with 0–9 points indicating absence of depression and ≥ 10 points was considered as presence of depression (53).

### Data collection procedures

Four BSc nurses served as data collectors, supervised by one MSc nurse. Prior to data collection, they underwent a one-day training session on the study’s objectives and conducting interviews using the standardized MMSE, CAGE and PHQ 9 tools. This training familiarized them with each question and techniques to minimize bias during data collection. Patients were informed in detail about the study’s purpose and significance, and their informed consent was obtained in writing. Data were collected through structured interviews using a questionnaire initially developed in English and translated into Amharic. Throughout the data collection process, the MSc nurse supervised to ensure completeness, accuracy, and consistency, with overall supervision by the principal investigator. The questionnaires (MMSE, CAGE and PHQ 9) were administered under uniform conditions in a quiet, well-lit room. Patients were instructed to answer each question carefully, and results were documented immediately. Additionally, data collectors recorded Patients’ last three blood glucose levels, any existing comorbidities, and blood pressure measurements from their medical charts as requested for the checkup.

### Data collection tools

Data on socio-demographic variables of DM patients were collected by interviewing using structured pretested Amharic version questionnaires adopted from a literature in Ethiopia. Data on behavioral variables were collected by using CAGE assessment tool which were validated and used by Ethiopian researchers (20). The CAGE assessment consists of four questions that address key behaviors and attitudes associated with alcohol use. Each question targets specific aspects: the desire to cut down on drinking, feelings of annoyance from others’ criticism, guilt about drinking habits, and the use of alcohol as an early morning “eye-opener.” Scoring is straightforward, with each “Yes” response indicating a potential problem. A total score of 2 or higher suggests a higher likelihood of alcohol misuse.

Data on cognition was collected by interview using a MMSE form which involves a related series of questions or commands which was previously used by the study in Ethiopia (42).

The MMSE consists 11 questions [30-point questionnaire test; orientation (10 points), registration (3 points), attention and calculation (5 points), recall (3 points), language and praxis [9 points; naming, repetition, 3-stage command, reading, writing and copying] (54).

Data on Depression was collected by interview using nine items of patient health questionnaire-9 (phq-9) PHQ-9 which involves a related series of questions which was previously used by the study in Ethiopia (55). The Depression scale ranges from 0 to 27 points, with higher score indicating Depression state. Depression was measure by using nine items of patient health questionnaire-9 (phq-9) PHQ-9 total score of the scale is 27 points, with 0–9 points indicating absence of depression and ≥ 10 points was considered as presence of depression (53). Patients’ charts were reviewed for blood glucose level, and for clinical related variables like, presence of comorbidities. The individual was received one point for each correct answer.

### Data quality control

To ensure the quality of the data, the questionnaires were first developed in English and then translated into Amharic. This translation was subsequently back translated into English by two independent language experts to maintain consistency. Data collectors participated in a two-day training session led by the principal investigator, which covered the study instruments, ethical research practices, and data collection techniques. Prior to the actual data collection, a pre-test was conducted with 5% of Patients at XXX Primary Hospital. Throughout both the pre-test and the main study, the supervisor and principal investigator closely monitored the daily data collection process. Five incomplete responses were discarded and recorded as non-response rates. During the analysis phase, the investigator reviewed the collected data to ensure its completeness. Additionally, establishing initial rapport with Patients before the formal interviews facilitated effective communication throughout the study.

### Data analyses techniques

The data were first checked for completeness and inconsistencies manually. Then enter into Epi-data version 4.6 and export to SPSS version 26 for processing and analysis. Necessary data processing like re-coding, categorizing, computing, and counting were done before the actual data analysis. The descriptive result was summarized as frequency tables, pie, and bar charts based on the type of data. The Binary logistic regression model was used to identify factors associated with NCI.

All variables with a p-value of less than 0.25 in the Bi variable logistic regression analysis after Model fitness was checked by Lemeshow goodness and Hosmer model and enter the multivariable logistic regression model to identify factors independently associated with NCI. Adjusted Odds ratios (AORs) with their corresponding 95% confidence intervals were used to assess the strength of associations between the outcome and predictor variables. The variables with p–value less than 0.05 was considered statistically significant.

## Results

### Socio demographic characteristics of the participants

A total of 512 participants were interviewed, yielding a response rate of 97%. The mean age of the participants was 44 ± 16 years, with ages ranging from 18 to 86 years. The age distribution revealed that 188 participants (36.7%) were between 30-45 years old, followed by 112 participants (21.9%) aged 46-60 years, and 110 participants (21.5%) aged 18-29 years. More than half of the participants, 300 (58.6%), were female. In terms of marital status, more than half of the participants, 338 (66%), were married. Educational background data showed that slightly over half of the participants, 282 (53.2%), had completed college education. Additionally, more than half of the participants, 298 (58.2%), resided in urban areas. Nearly three forth of the participants, 380 (74.2%), were Orthodox Christians (**Table 1**).

**Table 1:** Socio demographic characteristics of diabetes mellitus patients at referral hospital, northwest, Ethiopia March-April 2023(n=512).

Variables	Category	Frequency	Percent (100%)
Age in years	18-29	110	21.5
	30-45	188	36.7
	46-60	112	21.9
	>60	102	19.9
Sex	Male	212	41.41
	Female	300	58.59
Marital status	Single	123	24.0
	Married	338	66.0
	Divorce	34	6.6
	Widowed	16	3.1
	Separated	1	0.2
Residence	Urban	298	58.2
	Rural	214	41.8
Educational level	≤ Grade 8	117	22.8
	9-12 Grad	113	22.1
	College and above	282	53.2
Religion	Orthodox	380	74.2
	Muslim	106	20.7
	Protestant	26	5.1

**Figure 1:**
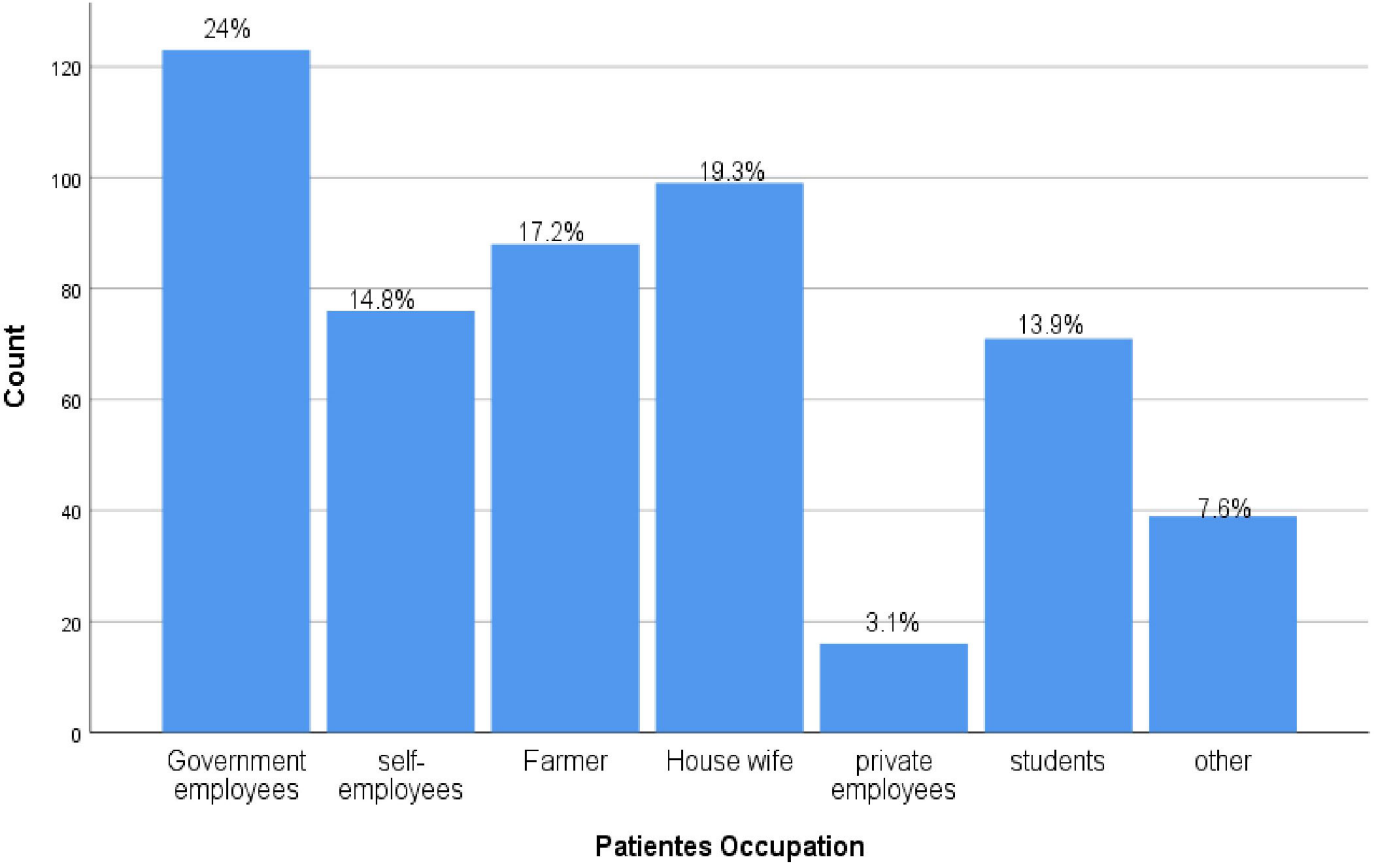
Occupation frequency from DM outpatients in Deber Markos referral hospital, Northwest, Ethiopia from March-April 2023(n=512).

Regarding occupation 123(24.04%) of study participants were government employers followed by Housewife 99(19.34%). NB: Others include Merchant, drivers, NGO workers, retired individuals (**Figure 1**).

### Clinical related factors of the participants

More than half of the participants, 292 (57.0%), were type 2 DM patients. The mean BMI was 24 ± 3 kg/m², with nearly half (53.5%) having a BMI in the 18.5-24.99 range. The mean weight was 67 ± 9 kg, and the median height was 168 ± 5 cm. The median duration of diabetes diagnosis was 5 ± 4 years, with more than half of the participants, 268 (52.3%), diagnosed within the past 1-5 years. Among the patients, 177 (34.57%) were taking only insulin whereas 105 (20.51) were taking both insulin and oral hypoglycemic agents and the rest 230 (44.92%) were taking only oral hypoglycemic agents. Regarding blood glucose levels, 47.3% of participants had levels within the normal range (70-126 mg/dl), while 46.1% had levels above the normal range. Additionally, 206 participants (40.2%) had a depression disorder (**Table 2**).

**Table 2:** Clinical related factors of diabetes mellitus patients at referral hospital, northwest, Ethiopia March-April 2023(n=512).

Variables	Category	Frequency	Percent (100%)
Types of DM	T1DM	220	43.0
	T2DM	292	57.0
Duration of diseases of DM	1-5years	268	52.3
	6-10years	137	26.8
	>10years	107	20.9
DM treatment modalities	Insulin only	177	34.57
	Oral hypoglycemic agents only	230	44.92
	Both	105	20.51
Co morbidities	No	302	59.0
	Yes	210	41.0
Types of co morbidities	Hypertension	158	80.61
	Chronic liver disease	15	7.65
	Cardiovascular disease	23	11.73
Body Mass Index	<18.5	19	3.7
	18.5-24.99	274	53.5
	>24.99	219	42.8
Serum glucose level	>126	236	46.1
	70-126	242	47.3
	<70	34	6.6
Depression	Yes	306	59.8
	No	206	40.2

**Figure 2:**
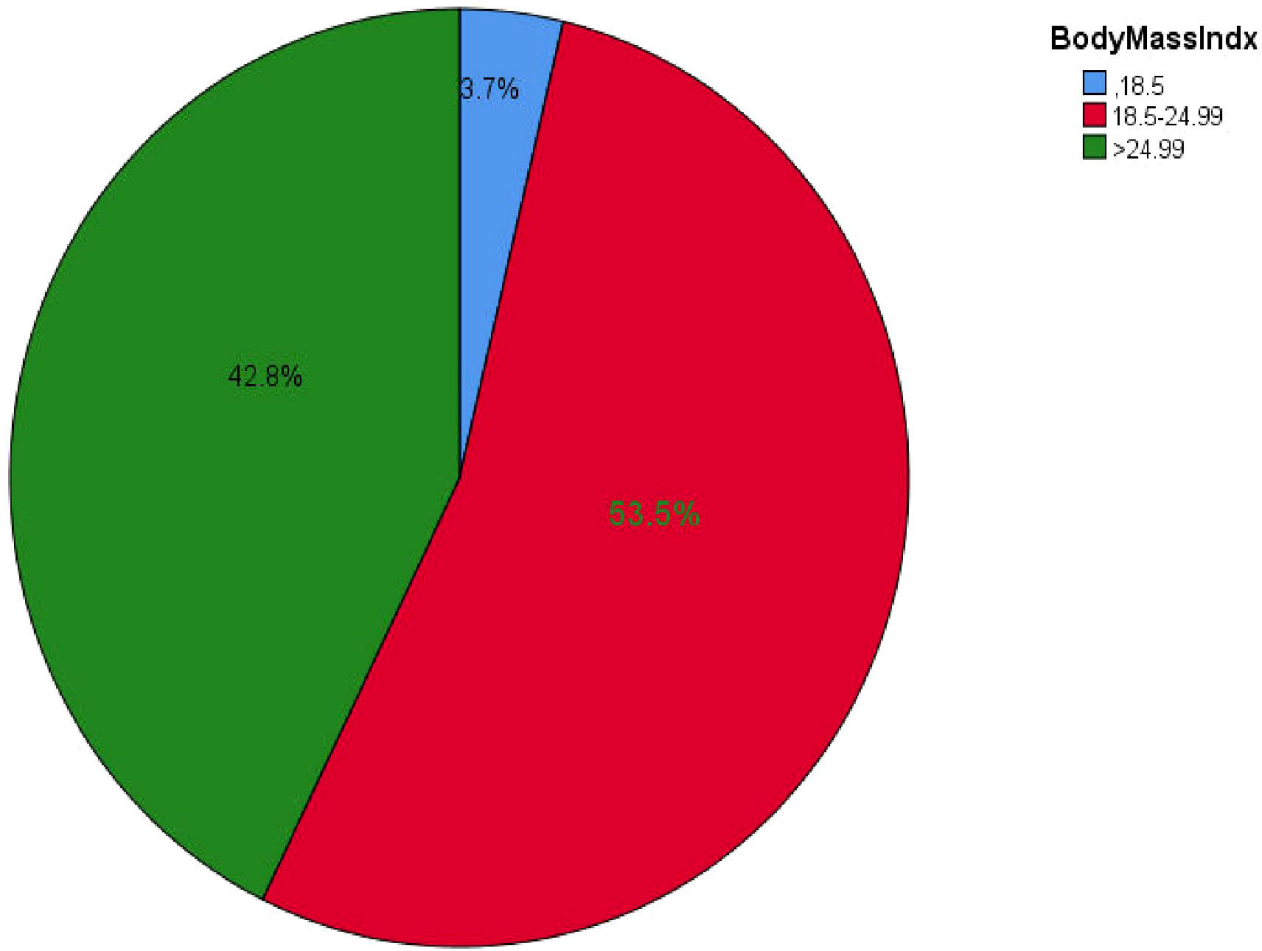
BMI of all types of DM outpatients in Debre Markos referral hospital, Northwest, Ethiopia from March-April, 2023(n=512).

The study results indicated that the median BMI of participants was 24 ± 3 kg/m², with values ranging from a minimum of 16 kg/m² to a maximum of 33.02 kg/m². Slightly more than half of the participants, 274 (53.5%), had a BMI within the normal range (**Figure 2**).

### Behavioral related factors of study participants

Among the participants, 9 (1.8%) had a history of smoking, with 4 (44.4%) of these individuals having a history of tobacco abuse. Additionally, 110 participants (21.5%) reported drinking alcohol after starting their medications, with 41 (37.7%) of them experiencing alcohol abuse. Furthermore, 15 patients (2.9%) had a history of chewing khat after starting their medications, with 5 (33.3%) being dependent on khat (**Table 3**).

**Table 3:** Behavioral related factors of diabetes mellitus patients at referral hospital, northwest, Ethiopia March-April 2023(n=512).

Variables	Category	Frequency	Percent (100%)
Smoking	No	503	98.2
	Yes	9	1.8
Tobacco abuse	No	5	55.6
	Yes	4	44.4
Alcohol drinking	No	402	78.5
	Yes	110	21.5
Alcohol abuse	No	69	62.7
	Yes	41	37.7
Chewing chat	No	497	97.1
	Yes	15	2.9
Chewing abuse	No	10	66.7
	Yes	5	33.3

### Prevalence of NCI among diabetes mellitus patients

Among the study participants, 145 were found to have cognitive impairment, resulting in an overall prevalence of 28.3% [95% CI: 24.57–32.39]. The median total MMSE score was 25, with an interquartile range of 5 points, and scores ranging from 20 to 30.

### Factors associated with neuro cognitive impairment among diabetes mellitus patients

In the bivariable analysis, twelve variables were selected for inclusion in the multivariable analysis: sex, age, occupation, residence, education, alcohol drinking status, body mass index, type of diabetes, duration of diagnosis, diabetes treatment modalities, comorbidities, and blood sugar level. Ultimately, the multivariable logistic regression analysis revealed that only five variables sex, comorbidity, blood sugar level, duration of disease diagnosis, and residence were significant factors for neurocognitive impairment among diabetic patients attending outpatient follow-ups.

Accordingly, the analysis revealed that female diabetes patients had 2.29 times higher risk of developing cognitive impairment compared to males [AOR=2.29, 95% CI: 1.43-3.67]. Similarly, patients living in rural areas had 3.16 times higher risk of cognitive impairment than those in urban areas [AOR=3.16, 95% CI: 2.01-4.95]. Patients with comorbidities faced 3.30 times higher risk of cognitive impairment than those without comorbidities [AOR=3.30, 95% CI: 2.08-5.23]. Additionally, those with blood sugar levels above 126 mg/dl had 2.25 times higher risk compared to patients with normal fasting blood glucose levels [AOR=2.25, 95% CI: 1.42-3.57]. Patients diagnosed 6-10 years ago had 1.72 times higher risk of cognitive impairment than those diagnosed less than 6 years ago [AOR=1.72, 95% CI: 1.01-2.94]. Similarly, patients diagnosed more than 10 years ago had 1.92 times higher risk than those diagnosed less than 6 years ago [AOR=1.92, 95% CI: 1.09-3.38] (**Table 4**).

**Table 4:** Multivariable logistic regression analysis to identify the predictors of neuro cognitive impairment among of diabetes mellitus patients at referral hospital, northwest, Ethiopia March-April 2023(n=512).

Variables	Category	NCI		COR (95% CI)	AOR (95%CI)	P-Value
		Yes	No			
Sex	Female	99	201	1.78 (1.19-2.67)	2.22 (1.39-3.55)	**0.001**
	Male	46	166	1	1	
Occupation	Self-employees	18	58	.88 (.454-1.716)	0.83 (0.37-1.86)	0.655
	Farmer	35	53	1.87 (1.04-3.38)	0.89 (0.36-2.19)	0.799
	Housewife	33	64	1.47 (0.82-2.62)	1.02 (0.45-2.29)	0.968
	Private employees	6	12	1.42 (0.49-4.01)	2.16 (0.41-11.49)	0.365
	Students	8	63	0.36 (0.16-0.84)	0.63 (0.17-2.28)	0.482
	Other	13	26	1.42 (0.65-3.10)	1.20 (0.46-3.15)	0.701
	Government employees	32	91	1	1	
Residence	Rural	89	125	3.078 (2.07-4.58)	3.11 (1.99-4.86)	**0.000**
	Urban	56	242	1	1	
Age in years	30-45	36	152	1.77 (0.89-3.50)	1.66 (0.76-3.65)	0.5
	46-60	47	65	5.40 (2.71-10.76)	2.44 (1.04-5.74)	0.9
	>60	49	53	6.90 (3.44-13.85)	2.60 (1.06-6.39)	0.7
	18-29	13	97	1	1	
Educational status	Education level ≤ 8	42	75	1.94 (1.22-3.12)	0.62 (0.33-1.15)	0.127
	Grade 9-12	40	73	1.90 (1.18-3.07)	0.74 (0.41-1.32)	0.307
	College &above	63	219	1	1	
Alcohol status	Yes	40	70	1.62 (1.03-2.53)	0.94 (0.54-1.64)	0.820
	No	105	297	1	1	
Types of Diabetic mellitus	T2DM	105	187	2.53 (1.66-3.84)	0.70 (0.34-1.42)	0.33
	T1DM	40	180	1	1	
Duration of diagnoses	6-10years	50	87	2.45 (1.54-3.88)	1.72 (1.01-2.94)	**0.045**
	>10years	44	63	2.97 (1.82-4.86)	1.92 (1.09-3.38)	**0.023**
	1-5years	51	217	1	1	
Diabetic mellitus treatment type	Oral hypoglycemic	75	155	2.28 (1.42-3.67)	1.19 (0.61-2.32)	0.602
	Both	39	66	2.78 (1.60-4.84)	1.48 (0.74-2.92)	0.266
	Insulin only	31	146	1	1	
Co morbidities	Yes	98	112	4.75 (3.14-7.17)	3.39 (2.14-5.36)	**0.000**
	No	47	255	1	1	
Body Mass Index	<18.5	6	13	1.75 (0.64-4.83)	1.96 (0.58-6.60)	0.28
	>24.99	82	137	2.28 (1.53-3.40)	1.35 (0.81-2.26)	0.247
	18.5-24.99	57	217	1	1	
Blood glucose level	<70	2	32	0.27 (0.06-1.18)	0.28 (0.06-1.26)	0.098
	>126	98	138	3.11 (2.05-4.71)	2.25 (1.42-3.57)	**0.001**
	70-126	45	197	1	1	

Bold values is ‘’significant factors’’.

## Discussion

The prevalence of neurocognitive impairment among diabetes mellitus patients at the referral hospital in Eastwest, Ethiopia, was found to be 28.3% [95% CI: 24.6–32.2]. This finding aligns with previous studies conducted in Ethiopia, including Bahirdar City referral hospitals (27.6%) (20), Addis Ababa 25% (56), as well as studies in Kenya (32%) (1) and and Poland (31.5%) (57).

On the other hand, this finding is higher than the 21.2% reported in a global systematic review study (30). The possible discrepancy may be due to differences in age adjustments and population characteristics. Similarly, the prevalence in this study was higher than that reported in Chile (17%) (35). The possible reason for this difference may be attributed to the setting of our study, which was conducted in a clinical setting, whereas in Chile, the study was conducted at the community level. Moreover, our study’s result was also higher than that of a study conducted in a tertiary care hospital in Vadodara, Gujarat, India (24%) (33). This discrepancy may be due to the cutoff point used for MMSE assessment in India (<23 MMSE indicating neurocognitive impairment) and the prospective follow-up design employed in the Indian study. These factors may have led to a lower prevalence of neurocognitive impairment compared to our cross-sectional study design.

However, the prevalence of neurocognitive impairment found in this study is lower than that reported in a study conducted in Jimma (53.3%) (58). This discrepancy may be attributed to differences in the ages of the study patients and modifications in the study population, which in our case included all diabetic patients rather than just those with type 2 diabetes mellitus. Similarly, the results of this study were lower than those reported in studies conducted in Egypt 34% (37), Nigeria 40% (38), Malaysia 46.9% (34), Saudi Arabia 80.3% (36) and various studies in India 33.73%, 54.3% and 50.5% (2, 20, 32). Furthermore, the prevalence found in this study was lower than that reported in systematic reviews and meta-analyses of mild cognitive impairment (MCI) (45.0%) (31), overall Europe 82.3% and Asia 98% (31). This discrepancy could be due to differences in the selection criteria of the study populations and sample sizes. The sample size in our study was smaller compared to those in the overall studies conducted in Europe and Asia.

In this study, being a female diabetic patient was identified as a significant risk factor for neurocognitive impairment compared to males. This finding is consistent with previous studies conducted in Saudi Arabia (36). The increased risk may be attributed to differences in body composition and fat distribution between genders, which can influence diabetic risk factors (59). Moreover, women with Type 2 diabetes are at a higher risk of experiencing accelerated cognitive decline than their male counterparts (60). Additionally, hormonal influences and psychosocial factors, such as stress and depression, may differentially impact cognitive health in men and women, contributing to this disparit (61).

Secondly, in this study, living in rural areas was associated with a 3.08 times higher risk of neurocognitive impairment compared to urban areas. This finding is consistent with a study conducted in India (33). This disparity may be attributed to factors such as lack of knowledge about diabetic complications, poor blood sugar control, late diagnosis of diabetes mellitus, and inadequate awareness of early screening for diabetes mellitus and other comorbidities.

Thirdly, the results of this study indicate that the presence of comorbidities is an independent risk factor for neurocognitive impairment, with patients having comorbidities being 4.75 times more likely to experience impairment compared to those without, across all types of diabetes mellitus. This finding aligns with studies conducted in Bahir Dar (20) and Poland (57). This increased risk may be attributed to immune deficiencies in patients with multiple diseases, which can adversely affect neural and cognitive functions (62). Among the comorbidities, hypertension contributes to cognitive decline through vascular mechanisms. Additionally, individuals with depression show higher odds ratios for developing cognitive impairment, and those with epilepsy frequently experience cognitive deficits (63–66).

Forthly, DM patients with a duration of diabetes between 6-10 years and over 10 years had neurocognitive impairment rates of 36.49% and 41.12%, respectively, compared to 19.01% in those with a duration of diabetes ≤5 years. The duration of diabetes between 6-10 years was associated with a 2.45 times higher risk of neurocognitive impairment compared to shorter durations of the disease.

Fivthly, DM patients with over 10 years of diabetes duration had a 2.97 times higher risk of neurocognitive impairment than those with ≤5 years of duration. These findings are consistent with studies conducted in Saudi Arabia (36) and Egypt (37). The association between neurocognitive impairment and longer duration of diabetes is attributed to the cumulative harmful effects of diabetes. Prolonged diabetes duration increases the likelihood of hyperglycemic events, leading to oxidative stress, glucose toxicity, and glutamate toxicity, which directly harm neurons and contribute to cognitive impairment. Additionally, hyperglycemia can lead to microvascular complications such as cerebral microangiopathy, further increasing the risk of neurocognitive impairment (36, 67).

Lastly, diabetic patients with blood sugar levels (BSL) above 126 mg/dL had a 3.11 times higher risk of neurocognitive impairment compared to patients with BSL between 70-126 mg/dL. This finding is consistent with a similar study conducted in Jimma (58). The increased risk is attributed to hyperglycemic events that trigger oxidative stress and lead to glucose and glutamate toxicity, directly damaging neurons. These factors contribute to cognitive impairment and can also cause microvascular complications, including cerebral microangiopathy, which further elevate the risk of neurocognitive impairment (36, 67).

### Strengths of the study

The cross-sectional design provides a snapshot of neurocognitive impairment prevalence among diabetic patients, facilitating comparisons across demographic and clinical factors. Using validated assessment tools ensures the findings are grounded in rigorous methodology. Furthermore, the focus on diabetic patients allows for a detailed examination of neurocognitive impairment, potentially leading to more targeted interventions.

### Limitations of the study

This study has several limitations, including the absence of imaging data, which hampers our ability to establish a direct relationship between diabetes, neuropathology, and cognitive deficits, primarily due to resource constraints. Additionally, the analysis treated comorbidities as a general variable rather than considering them as separate factors, which may affect the accuracy of our findings.

## Conclusion

Our study revealed a higher prevalence of NCI among all types of diabetic patients, affecting approximately one-fourth based on MMSE scores. Factors such as female gender, rural residence, comorbidities, longer diabetes duration, and elevated blood glucose levels were identified as significant risks contributing to increased prevalence of NCI among diabetic patients. Identifying and addressing modifiable factors that influence cognitive impairment in this population is crucial.

## Recommendations

DM is strongly associated with neurocognitive impairment, which can be exacerbated by disease severity. Therefore, it is essential for patients to adhere to medication regimens for proper blood glucose control. Healthcare professionals should educate patients on sugar-free diets, routine diabetes care, early detection of comorbidities, and regular neurocognitive function assessments tailored to patient understanding.

Patients with comorbidities are particularly at risk and should undergo regular neurocognitive assessments as part of their diabetes management. Education on diabetes complications is crucial, especially for rural residents who may lack awareness. Universal diabetes screening based on blood glucose levels should be implemented at community levels by regional health bureaus and the Ministry of Health.

Strategies focused on early detection of both diabetes and cognitive impairment, using tools like MMSE, should be developed. Accessible, affordable, and comprehensive diabetes care services must include cognitive status assessments. Researchers should further explore specific types of NCIs and their associated risk factors among adults with diabetes using advanced diagnostic techniques such as MRI or CT scans.

These measures aim to improve early detection, management, and prevention of diabetes-related complications, thereby enhancing overall patient outcomes and quality of life.

We strongly recommend that future researchers employ a robust study design and treat comorbidities as separate variables in their analyses.

## Data Availability

The raw data supporting the conclusions of this article will be made available by the authors, without undue reservation.
